# The glyoxylate cycle and alternative carbon metabolism as metabolic adaptation strategies of *Candida glabrata*: perspectives from *Candida albicans* and *Saccharomyces cerevisiae*

**DOI:** 10.1186/s12929-019-0546-5

**Published:** 2019-07-13

**Authors:** Shu Yih Chew, Wallace Jeng Yang Chee, Leslie Thian Lung Than

**Affiliations:** Department of Medical Microbiology and Parasitology, Faculty of Medicine and Health Sciences, Universiti Putra Malaysia, 43400 UPM Serdang, Selangor Malaysia

**Keywords:** *Candida*, *Candida glabrata*, Carbon metabolism, Glyoxylate cycle, Metabolic adaptation, Pathogenesis, virulence, yeast.

## Abstract

**Background:**

Carbon utilization and metabolism are fundamental to every living organism for cellular growth. For intracellular human fungal pathogens such as *Candida glabrata*, an effective metabolic adaptation strategy is often required for survival and pathogenesis. As one of the host defence strategies to combat invading pathogens, phagocytes such as macrophages constantly impose restrictions on pathogens’ access to their preferred carbon source, glucose. Surprisingly, it has been reported that engulfed *C. glabrata* are able to survive in this harsh microenvironment, further suggesting alternative carbon metabolism as a potential strategy for this opportunistic fungal pathogen to persist in the host.

**Main text:**

In this review, we discuss alternative carbon metabolism as a metabolic adaptation strategy for the pathogenesis of *C. glabrata*. As the glyoxylate cycle is an important pathway in the utilization of alternative carbon sources, we also highlight the key metabolic enzymes in the glyoxylate cycle and its necessity for the pathogenesis of *C. glabrata*. Finally, we explore the transcriptional regulatory network of the glyoxylate cycle.

**Conclusion:**

Considering evidence from *Candida albicans* and *Saccharomyces cerevisiae*, this review summarizes the current knowledge of the glyoxylate cycle as an alternative carbon metabolic pathway of *C. glabrata*.

## Introduction

Fungi are a group of eukaryotic organisms that can provoke a broad range of infectious diseases in humans, ranging from mild superficial infections to potentially life-threatening systemic infections. *Candida* species, such as *Candida albicans* and *Candida glabrata,* are ubiquitous and are considered benign residential microflora in the human body. However, an increased presence of immunocompromised individuals in human populations can contribute to the spread of candidiasis infections [1–3]. Invasive candidiasis is common among intensive care unit (ICU) and organ transplant patients and is often associated with increased costs of care, significant reductions in lifespan and prolonged hospitalization even after receiving antifungal treatments [4–8].

Bloodstream infections (BSIs) caused by *Candida* species or candidaemia are the most common manifestation of invasive candidiasis. In recent years, a shift of predominance from *C. albicans* to non-*C. albicans Candida* (NCAC) species has been observed, and *C. albicans* now accounts for only half of the invasive candidiasis cases reported [9]. Notably, *C. glabrata* has been recognized as either the second or third most common cause of invasive candidiasis after *C. albicans* [10], and similar epidemiological data have also been highlighted in global surveillance programmes such as SENTRY, ARTEMIS and TRANSNET [11–14]. Pfaller et al. (2014) reported that invasive candidiasis caused by *C. glabrata* is most commonly associated with patients who receive treatment for solid tumours or undergo solid organ transplantation (SOT) [15]. Among the patients with solid tumours and infections caused by NCAC species, 53.8% were caused by *C. glabrata*. In addition, *C. glabrata* was the main aetiological agent of 63.7% of SOT patients with NCAC infections. Overall, the increasing prevalence of NCAC species, particularly *C. glabrata,* is alarming due to the high mortality rates associated with such infections; thus, there is an urgent need to improve the current diagnostic and therapeutic options for better management of fungal BSIs [16].

Host immunity was believed to be the main factor responsible for the establishment of opportunistic fungal infections caused by yeast pathogens. However, this concept has changed, and it is now agreed that the pathogenicity of these yeast pathogens also plays a crucial role in infection [17]. For instance, virulence factors and fitness attributes such as metabolic flexibility, adherence to the host epithelia, biofilm formation, phenotypic switching, filamentation, secretion of hydrolytic enzymes, production of haemolysin and production of extracellular hydrolase are all associated with *Candida* species [18, 19]. Relative to *C. albicans*, the virulence factors and fitness attributes of *C. glabrata* are not well known, particularly in its natural niche.

The invasion strategy of *C. glabrata* is believed to be independent of phenotypic switching, as opposed to *C. albicans*, which employs invasion mechanisms that involve active penetration through hyphae extension and induced endocytosis [20, 21]. Despite being a relatively less virulent *Candida* species and generally having a weaker tissue-damaging ability compared to *C. albicans, C. glabrata* is still able to breach the natural barriers, invade the human bloodstream and cause systemic infection, presumably through SOT, parenteral nutrition, trauma, surgical or indwelling medical devices [8, 22]. In addition, *C. glabrata* uses a co-infection approach in infecting the human bloodstream, often by exploiting the tissue invasion and damage of the natural barriers caused by other *Candida* species, such as *C. albicans* [21, 23, 24].

Upon entering the human bloodstream, *C. glabrata* triggers a weaker polymorphonuclear neutrophil activation that primarily induces the recruitment of monocytic cells to the site of infection and triggers monocyte engulfment [25]. Since *C. glabrata* can survive and propagate within macrophages but not in neutrophils [26, 27], this fungal pathogen could use monocytes or macrophages as “Trojan horses” to gain protection against host defence mechanisms, particularly against neutrophil attack [25, 26]. In fact, *C. glabrata* clearly pursues different immune evasion and persistence strategies compared to *C. albicans*, as escape from the macrophages upon engulfment is not the priority of this fungal pathogen. Unlike *C. albicans*, *C. glabrata* can persist and replicate within macrophages without causing any significant damage to the host until the host cells eventually burst and release the fungal cells [26].

While trapped within macrophages, *C. glabrata* must rely on endogenous resources for survival because macrophages are often depicted as glucose-deficient [28, 29]. It has been shown that the ability of *C. glabrata* to mobilize intracellular resources through autophagy serves as a major contributor to sustaining the viability of this pathogen during carbon starvation [30, 31]. In addition to recycling intracellular resources via autophagy, the ability to utilize carbon sources other than glucose could potentially assist in the survival of engulfed *C. glabrata*. This review aims to discuss the metabolic adaptation and alternative carbon metabolism in *C. glabrata.* Taking cues from *Saccharomyces cerevisiae* and *C. albicans*, this review also discusses the role of the glyoxylate cycle in alternative carbon metabolism as well as the transcriptional regulation of this anabolic pathway in *C. glabrata.*

### Metabolic adaptation strategy in *C. glabrata*

Nutrients are crucial for all living organisms, including fungi. Since *Candida* species often inhabit host niches with different nutrient availability, these fungal pathogens must be equipped with a high degree of metabolic flexibility and possess metabolic adaptation mechanisms that are required for effective nutrient acquisition [32]. Despite being a common human pathogen and rarely being found in environmental niches, *Candida* species such as *C. albicans* and *C. glabrata* still retain an impressive degree of metabolic flexibility [33, 34]. In addition, the virulence factors and fitness attributes of these fungal pathogens are firmly dependent on fungal metabolism, strengthening the truism of “you are what you eat” [35].

Transcriptional analyses of macrophage-engulfed *C. albicans* and *C. glabrata* revealed extensive metabolic reprogramming that reflects nutrient deprivation [28, 29]. These effects include the upregulation of genes that encode key metabolic enzymes in three interconnected anabolic pathways for alternative carbon metabolism, i.e., gluconeogenesis (*PCK1* and *FBP1*), glyoxylate cycle (*ICL1* and *MLS1*) and β-oxidation of fatty acids (*FOX2*, *POT1* and *POX1*), coupled with the downregulation of genes related to glycolysis and translational machinery (e.g., elongation factors, ribosomal protein genes, translation initiation and tRNA synthetases). The induction of these key enzyme genes suggests that the microenvironment within macrophages is deficient in glucose; however, the confined *C. glabrata* cells are still able to utilize endogenous resources such as alternative carbon sources for their growth and survival. Furthermore, Rai et al. (2012) reported that engulfed *C. glabrata* cells primarily utilize acetyl coenzyme A (acetyl-CoA) from the breakdown of fatty acids via the glyoxylate cycle as the main carbon source for the production of cellular building blocks [36].

Other than macronutrients, fungal pathogens are also dependent on micronutrients for the full functionality of proteins and enzymes, which are essential for survival and host colonization [32]. Trace metals such as iron are usually sequestered from fungal pathogens via nutritional immunity, forcing these fungal pathogens to employ iron acquisition mechanisms during iron starvation. In *Candida* species, *C. albicans* is able to obtain xenosiderophores from other microorganisms through iron parasitism using the Sit1 siderophore transporter [37]. Iron parasitism and the Sit1 transporter are well conserved in many fungal species, such as *S. cerevisiae*, *Cryptococcus* and *Aspergillus* [38]. Nevertheless, *C. glabrata* is unable to use siderophores of bacterial origin for iron uptake. In fact, *C. glabrata* can only exploit fungal xenosiderophores via the Sit1 homologue transporter, which is critical for its survival in macrophages [39]. To date, the uptake of other important trace metals in *C. glabrata*, such as copper and manganese, remains uncharacterized.

In addition to reprogramming its metabolic activity, *C. glabrata* obtains nutrients through mobilization of intracellular resources via a specialized autophagy known as pexophagy. Deletion of the genes required for pexophagy, *ATG11* and *ATG17*, rendered *C. glabrata* more susceptible to macrophage killing. Therefore, pexophagy could potentially assist trapped *C. glabrata* in overcoming transient periods of nutrient deprivation in the phagosome and facilitate disseminated infection [30].

### Alternative carbon metabolism in *C. glabrata*

Glucose remains the preferred carbon source for *Candida* species, and it is an important carbon and energy source for cell growth and development. Generally, glucose sensing and uptake in *Candida* species involve three main pathways, including the sugar receptor-repressor pathway, adenylate cyclase pathway and glucose repression pathway [40]. However, the availability of glucose varies in many host niches and anatomical sites, ranging from 0.05–0.1% in vaginal secretion to 0.1–0.2% in the blood [41, 42]. Apart from glucose, yeast cells are able to utilize alternative carbon sources, including but not limited to lactate, acetate, ethanol, glycerol and oleate [43]. Organic and short-chain fatty acids (SCFAs), such as acetate and lactate, are produced by intestinal bacteria from the degradation of complex carbohydrates [42–44]. Acetate and lactate can also be found in other host niches, such as the vaginal environment, which is dominated by vaginal microflora. Previously, it has been shown that *C. glabrata* can survive in carbon starvation conditions upon macrophage engulfment [30]; therefore, it is likely that alternative carbon metabolism plays a major role in the survival of *C. glabrata* trapped within macrophages.

**Fig. 1:**
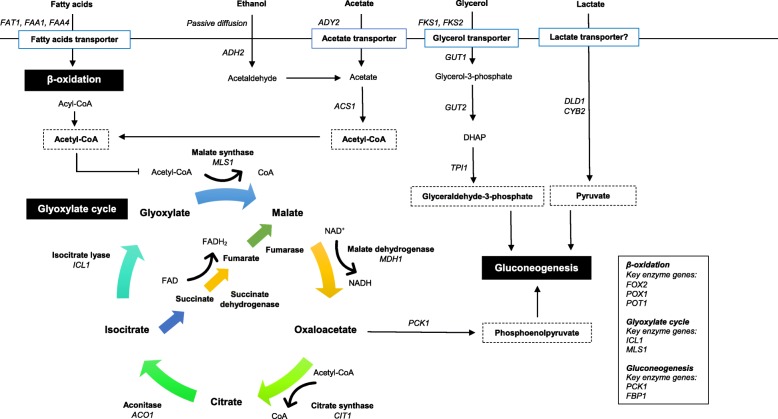
Schematic representation of glyoxylate cycle and its involvement in alternative carbon metabolism. In the absence of glucose, alternative carbon sources acids are transported and utilized by *C. glabrata*, converted to the central metabolite acetyl-CoA to fuel glyoxylate cycle and gluconeogenesis for glucose and energy production [23, 38, 52, 53]

Glycerol is utilized by yeast cells as an important carbon source as well as for osmoregulation [45]. In fact, glycerol is also one of the alternative carbon sources that can be utilized by *C. glabrata*, and the uptake process is probably mediated by the glycerol transporter encoded by *FPS1* and *FPS2* [46]. Glycerol is first converted to glycerol-3-phosphate by the glycerol kinase Gut1, followed by conversion to dihydroacetone phosphate (DHAP), catalysed by glycerol-3-phosphate dehydrogenase (Fig. 1). Subsequently, DHAP can enter gluconeogenesis through conversion into glyceraldehyde 3-phosphate by triose phosphate isomerase. On the other hand, lactate is transported through a monocarboxylate permease encoded by *JEN1* in yeast such as *S. cerevisiae* and *C. albicans*. The presence of glucose represses the expression of *JEN1,* and the degradation of protein-coding mRNA is also observed when induced *JEN1* is exposed to glucose [47, 48]. Lactate is converted to pyruvate by two different oxidoreductases encoded by *CYB2* and *DLD1* [49], which serves as an intermediate for gluconeogenesis. Although *JEN1* is absent in *C. glabrata* [50], lactate dehydrogenase-mediated lactate assimilation has been shown to be vital for the survival and adaptation of *C. glabrata* in the mouse intestine [51].

Ethanol is believed to enter the cells through passive diffusion, whereas acetate is transported to the cells through the carboxylate transporter acetate permease [54–56]. Both carbon sources are ultimately converted to acetyl-CoA by acetyl-CoA synthetase (Acs1) in the cytosol (Fig. 1). It has been shown that *C. glabrata* are able to utilize acetate, even in the presence of glucose [57, 58]. In addition, the putative acetate permease gene *ADY2* was induced in *C. glabrata* in response to engulfment by macrophages [29] and neutrophils [59]. These observations suggest that acetate is relevant in vivo and that *C. glabrata* may assimilate acetate or other SCFAs in microenvironments with poor glucose availability. In addition, transporters such as Fat1, Faa1 and Faa4 are believed to be involved in the uptake of fatty acids in yeast cells [60]. Orthologous genes that encode these fatty acid transporters have been identified in *C. glabrata*, although they have not been fully characterized. Unlike ethanol and acetate, fatty acids are broken down to acetyl-CoA via β-oxidation in the peroxisomes, which involves the enzymes fatty acyl-CoA oxidase, 3-hydroxyacyl-CoA dehydrogenase and 3-oxoacyl-CoA thiolase. Acetyl-CoA generated from the breakdown of acetate, ethanol and fatty acids fuels the glyoxylate cycle and gluconeogenesis for glucose production when glucose availability is scarce (Fig. 1) [28, 43].

The breakdown of alternative carbon sources, such as fatty acids, ethanol and acetate, leads to the generation of the central metabolite acetyl-CoA in different subcellular compartments [52]. Specifically, breakdown of fatty acids generates acetyl-CoA that is solely peroxisomal, while breakdown of ethanol and acetate produces cytosolic acetyl-CoA. Due to its bulkiness and amphiphilic nature, transport of peroxisomal and cytosolic acetyl-CoA to the glyoxylate cycle often requires a functional acetyl unit transport system [52, 61]. In general, *S. cerevisiae* appears to use two parallel modes of acetyl unit transportation systems, namely, the carnitine shuttle and citrate synthase pathway, while *C. albicans* exclusively depends on the carnitine shuttle [reviewed in [52]]. The genome of *C. glabrata* also encodes three still uncharacterized, putative carnitine acetyltransferase orthologues, Cat2, Yat1 and Yat2, which are similar to those in *S. cerevisiae* and *C. albicans* [62, 63], suggesting that a functional carnitine shuttle also exists in *C. glabrata* for acetyl-CoA transportation. It has been shown that *C. albicans* is fully dependent on the carnitine shuttle for transportation of acetyl-CoA due to the absence of peroxisomal citrate synthase (Cit2), and this is further underlined by the fact that *C. albicans* also possesses a complete carnitine biosynthesis pathway [64–66]. In contrast to *C. albicans*, a putative Cit2 orthologue exists in *C. glabrata* (60% similarity to *S. cerevisiae* Cit2), suggesting that *C. glabrata* may utilize two modes of acetyl unit transportation, similar to the phylogenetically more closely related *S. cerevisiae*. Nonetheless, further investigation is needed to fully decipher the carnitine shuttle and potential peroxisomal citrate synthase pathway in *C. glabrata*.

### Glyoxylate cycle: an overview

The glyoxylate cycle is an anaplerotic variant of the tricarboxylic acid (TCA) cycle and is an anabolic pathway occurring in most protists, plants, bacteria and fungi. The glyoxylate cycle is assumed to be absent in animals and human tissues, with nematodes at the early stages of embryogenesis being the only known exception [67, 68]. The primary function of the glyoxylate cycle is to allow growth when glucose is not available and two-carbon compounds, such as ethanol and acetate, are the only sources of carbon [69]. The glyoxylate cycle is basically a shunt in the TCA cycle, with which it shares many metabolic enzymes, including malate dehydrogenase, citrate synthase, and aconitase [70]. The two decarboxylation steps involve isocitrate dehydrogenase and α-ketoglutarate dehydrogenase in the TCA cycle and are essentially excluded from the glyoxylate cycle. Alternatively, the glyoxylate cycle contains two additional key enzymes: isocitrate lyase and malate synthase (Fig. 1). In brief, isocitrate lyase catalyses the breakdown of isocitrate (C_6_) into glyoxylate (C_2_) and succinate (C_4_), followed by condensation of glyoxylate with acetyl-CoA (C_2_) catalysed by malate synthase to generate malate (C_4_) and a free CoA (Fig. 1). Therefore, malate produced from C_2_ compounds (e.g., ethanol and acetate) can serve as an intermediate to replenish the TCA cycle in the absence of glucose [71]. In addition, malate serves as the precursor of oxaloacetate, an essential substrate for gluconeogenesis. Oxaloacetate is converted to phosphoenolpyruvate by the gluconeogenesis enzyme phosphoenolpyruvate carboxykinase and eventually leads to glucose production with the help of another gluconeogenesis enzyme, fructose-1,6-biphosphate (Fig. 1) [71]. In short, the glyoxylate cycle bypasses the two decarboxylation steps in the TCA cycle to allow for the anabolism of simpler carbon compounds in gluconeogenesis.

The glyoxylate cycle and its key metabolic enzymes, isocitrate lyase and malate synthase, are believed to be highly conserved across different organisms [67, 72]. The glyoxylate cycle plays an important role in the growth of plant seedlings and is involved in the conversion of stored lipids to carbohydrates, which serve as the main nutritional resource for the plant before the commencement of photosynthesis [73]. The glyoxylate cycle is also well studied in bacterial pathogens, especially in *Mycobacterium tuberculosis*. Honer Zu Bentrup et al. (1999) showed that *M. tuberculosis* possesses a second copy of the functional *ICL1* gene (*aceA*) [53]. The glyoxylate cycle is reported to be essential for *M. tuberculosis* survival in the host because deletion of *ICL1* and *aceA* genes leads to complete impairment of intracellular replication and rapid elimination of this pathogen from the lungs of C57BL/6 mice in vivo [74]. In addition, the glyoxylate cycle is essential for the pathogenicity of other intracellular bacterial pathogens, namely, *Salmonella enterica* serovar Typhimurium, *Rhodococcus equi* and *Pseudomonas aeruginosa* [75–77].

Inactivation of the *ICL1* gene in the fungus *Leptosphaeria maculans*, a causal agent of the blackleg of crucifers, causes low germination rates of pycnidiospores, thus reducing the pathogenicity of this fungal pathogen on canola cotyledons [78]. In addition, the glyoxylate cycle is required for the full virulence of *Magnaporthe grisea*, the rice blast fungus. Disruption of *ICL1* in *M. grisea* causes a reduction in appressorium formation, conidiogenesis and cuticle penetration, leading to an overall decrease in damage to rice and barley leaves [79]. Furthermore, disruption of *MLS1* in the glyoxylate cycle also causes a reduction in the pathogenicity of certain plant pathogenic fungi, such as *Rhodococcus fascians*, *Stagonospora nodorum* and *Xanthomonas campestris* [80–82]. For intracellular human fungal pathogens, upregulation of glyoxylate cycle genes has been observed in *Paracoccidioides brasiliensis*, *Penicillium marneffei*, *C. albicans*, *A. fumigatus* and *C. neoformans* in response to macrophages [28, 83–86]. While the glyoxylate cycle is required for the full virulence of *C. albicans*, it is not essential for the full virulence of *A. fumigatus* and *C. neoformans* in vivo [87–89].

### Glyoxylate cycle and the virulence of *C. glabrata*: what we know so far

The glyoxylate cycle enzymes isocitrate lyase and malate synthase are highly similar in *C. glabrata* and *S. cerevisiae*, with protein sequence identities of approximately 84 and 86%, respectively. In contrast, both enzymes of *C. glabrata* and *C. albicans* have protein identities between 58 and 68%, respectively. The subcellular localizations of isocitrate lyase and malate synthase have been inferred in the peroxisomes of *C. albicans* [90]. However, the isocitrate lyase of *S. cerevisiae* is localized in the cytosol, while malate synthase is compartmentalized in both the peroxisomes and the cytosol [70]. For *C. glabrata*, the subcellular localizations of both enzymes still remain to be determined, although WoLF PSORT predicts that these enzymes are localized in the peroxisomes, similar to *C. albicans* [91]. This result implies that *C. glabrata* could potentially regulate the localization of isocitrate lyase and malate synthase in a way that is similar to *C. albicans*, albeit with glyoxylate cycle enzymes that resemble those of baker’s yeast.

The glyoxylate cycle in *Candida* species has been mainly studied in *C. albicans*, the predominant *Candida* species that causes invasive candidiasis in humans. Lorenz and Fink (2001) reported that the glyoxylate cycle genes *ICL1* and *MLS1* were significantly upregulated in *C. albicans* that were grown in the presence of macrophages [87]. In addition, the *ICL1* gene is required for the full virulence of this fungal pathogen. Homozygous deletion of *ICL1* rendered *C. albicans* avirulent in an in vivo mouse model of invasive candidiasis, while reintroducing *ICL1* restored the virulence of this fungal pathogen in vivo. Genetic characterization of the glyoxylate cycle genes *ICL1* and *MLS1* revealed high homologies to other fungal species, particularly *S. cerevisiae* and *C. tropicalis* [92]. In addition, *ICL1* and *MLS1* were repressed in the presence of glucose and only induced in the presence of alternative carbon sources, which is very similar to the regulation of the glyoxylate cycle in *S. cerevisiae*.

Global gene expression analysis of *C. albicans* showed that *ICL1* and *MLS1* were also highly upregulated (> 20-fold) in response to human blood [93], which is in concordance with the data obtained from a previous study [87]. In 2004, transcriptional profiling of *C. albicans* in response to murine macrophages was investigated by using a microarray approach [28]. Microarray analyses revealed a reprogramming event in *C. albicans* in response to nutrient starvation in macrophages. In fact, genes related to alternative carbon metabolism, including genes from gluconeogenesis, glyoxylate cycles and β-oxidation of fatty acids, were found to be induced upon macrophage engulfment.

Barelle et al. (2006) revisited the role of the glyoxylate cycle in the virulence of *C. albicans* by using a green fluorescent protein (GFP) fusion approach instead of transcript profiling, which allows for the monitoring of individual *C. albicans* within host niches [94]. Indeed, *ICL1*-GFP fusion was repressed at physiologically relevant glucose concentrations (0.1%) and only expressed in the presence of amino acids. Furthermore, induction of *ICL1*-GFP was observed in phagocytosed *C. albicans* in neutrophils and macrophages, but not in the non-phagocytosed cells. While most of the *C. albicans* cells expressed glycolytic genes *PYK1* and *PFK2* in the infected mouse kidneys, only half of the *C. albicans* cells expressed *ICL1*-GFP in the early stage of infection. This result implies that *ICL1* is essential for the virulence of *C. albicans* in the early stage of infection in response to macrophages. However, a glycolytic mechanism is required for the disease progression of invasive candidiasis in the later stage of infection.

In addition, Ramírez and Lorenz (2007) reported that mutants lacking the glyoxylate cycle enzyme gene *ICL1* displayed more extensive growth defects than initially predicted [95]. In addition to fatty acids, ethanol and acetate, *ICL1* mutants of *C. albicans* are unable to grow in medium with citrate or glycerol as the sole carbon source. This finding suggests that the regulation of alternative carbon metabolism in *C. albicans* may be different compared to other fungi, such as *S. cerevisiae*. Ramírez and Lorenz (2007) also reconfirmed that *ICL1* is required for the virulence of *C. albicans* by using a newly engineered strain that accounts for the positional effects of the *URA3* disruption marker used in a previous study [87, 96]. Fernández-Arenas et al. (2007) used a proteomic approach to show that the glyoxylate cycle in *C. albicans* is upregulated in response to challenges from murine macrophages [97]. The glyoxylate cycle enzymes isocitrate lyase and malate synthase are localized to the peroxisome in a Pex5p-dependent manner. However, peroxisomal localizations of these enzymes are not essential for the functioning of the glyoxylate cycle, as this metabolic pathway works equally well in the cytosol of the cells [90].

Sandai et al. (2012) reported that there is significant disagreement between the proteome and transcriptome profiles of *C. albicans* [98]. Although the presence of glucose triggers the degradation of the *ICL1* transcript, isocitrate lyase is stable and not destabilized, in contrast to the rapid destabilization of isocitrate lyase in *S. cerevisiae* following exposure to glucose. In fact, the isocitrate lyase of *C. albicans* lacks the main ubiquitination sites essential for targeted degradation of the transcript. These findings suggest that *C. albicans* underwent post-translational rewiring as it co-evolved with its host and thereby gained the ability to utilize alternative carbon sources and glucose simultaneously during systemic infection. Childers et al. (2016) investigated the impact of ubiquitin-mediated catabolite inactivation on the virulence of *C. albicans* [99]. The addition of ubiquitination sites in *C. albicans* leads to the destabilization of isocitrate lyase in response to glucose. Reduced metabolic flexibility following the addition of a ubiquitination site rendered *C. albicans* unable to use alternative carbon sources, more susceptible to macrophage killing, and unable to cause systemic and gastrointestinal tract infections in vivo.

As for *C. glabrata*, Kaur et al. (2007) have reported that the transcriptional response of *C. glabrata* co-incubated with macrophages is very similar to the response described in *C. albicans* [29]. Genes involved in gluconeogenesis, the glyoxylate cycle, β-oxidation of fatty acids and the methylcitrate cycle were induced upon macrophage engulfment. In addition, Rai et al. (2012) revealed that alternative carbon remained the main intracellular carbon source in macrophages, and *C. glabrata* utilized acetyl-CoA via the glyoxylate cycle and gluconeogenesis for energy production [36]. Our previous work showed that the *ICL1* gene is indeed crucial for the metabolism of alternative carbon sources in *C. glabrata* [100]. In fact, disruption of *ICL1* rendered *C. glabrata* unable to utilize acetate, ethanol or oleic acid as the sole carbon source for growth and survival. In addition, *ICL1* is also required for the intracellular survival of *C. glabrata* upon macrophage engulfment, potentially through the utilization of alternative carbon sources found within macrophages. Most importantly, *ICL1* is essential for the virulence of *C. glabrata* in vivo, as disruption of *ICL1* confers a severe attenuation in the virulence of *C. glabrata* in the mouse model of invasive candidiasis.

### Regulation of the glyoxylate cycle

Most of the transcriptional regulators in yeast belong to a subclass of proteins known as zinc cluster proteins. These proteins are characterized by a well-conserved motif (CysX2CysX6CysX5–12CysX2CysX6–8Cys) and are fungal specific [101]. As most zinc cluster proteins bind to DNA, they are essential for the regulation of yeast transcriptional and translational machineries [102]. To date, transcriptional regulation of alternative carbon metabolism has not been well studied in *C. glabrata*. In *S. cerevisiae*, Snf1 kinase is the main activator of downstream glucose-repressed genes and is important for the activation of alternative carbon metabolism. Generally, Snf1 kinase is induced in response to glucose-deficient conditions and deactivates the transcriptional repressor Mig1 by phosphorylation [43]. Dissociation of Mig1 from the co-repressor protein complex Ssn6-Tup1 leads to increased expression of Cat8, a transcriptional regulator that binds the carbon source-responsive element (CSRE) located at the promoter region of many glyoxylate cycle and gluconeogenic genes such as *ICL1*, *MLS1*, *FBP1* and *PCK1* [43, 103, 104].

Cat8 is also an activator of Sip4, another important CSRE-binding transcriptional regulator found in *S. cerevisiae*. Although Cat8 and Sip4 have similar functions in regulating the glyoxylate cycle and gluconeogenic genes, Cat8 appears to be the primary regulator, because the removal of Cat8 significantly reduced the expression of CSRE-dependent genes, while the absence of Sip4 only caused a minor reduction in the expression of these genes [105, 106]. Furthermore, activation of Sip4 requires a functional *CAT8*, whereas activation of Cat8 is not dependent on *SIP4*. Unlike Mig1-regulated Cat8, activation of Sip4 is regulated by Snf1 Kinase and Rds2, another transcriptional regulator [43, 107]. Like Cat8 and Sip4, Rds2 also binds to the promoter region of CSRE-dependent genes from the glyoxylate cycle and gluconeogenesis following exposure to alternative carbon sources [107]. In short, CSRE-dependent genes from the glyoxylate cycle are at least recognized and regulated by three transcriptional regulators, namely, Cat8, Sip4 and Rds2, in *S. cerevisiae*.

Interestingly, removal of *CAT8* did not alter the expression of CSRE-dependent genes that encode enzymes for the glyoxylate cycle, gluconeogenesis and β-oxidation of fatty acids in *C. albicans* [108]. Additionally, disruption of *CAT8* did not lead to any noticeable attenuation in the virulence of *C. albicans* in vivo. This result suggests that Cat8 is not involved in the transcriptional regulation of glyoxylate cycle genes and is also not required for the full virulence of *C. albicans*. This observation could potentially be attributed to the substantial reassignment of the function of some transcriptional regulators between *C. albicans* and *S. cerevisiae*, which are reported to be highly divergent [109]. Therefore, it remains to be seen whether the regulation of the glyoxylate cycle in *C. glabrata* follows the more phylogenetically related *S. cerevisiae* or other *Candida* species from the CTG clade, such as *C. albicans*. Moreover, orthologous genes of *SIP4* and *RDS2* have been found in both *C. albicans* and *C. glabrata* [110, 111]; however, their involvement in the regulation of alternative carbon metabolism, particularly the glyoxylate cycle, remains unclear.

## Conclusion

In conclusion, rapid and effective metabolic adaptation within the host is crucial for human fungal pathogens to thrive. The present review discussed the importance of alternative carbon metabolism as a metabolic adaptation strategy for intracellular *C. glabrata* to survive and propagate in carbon starvation. Considering the essential role of the glyoxylate cycle, particularly *ICL1,* in the virulence of *C. glabrata*, further studies should be conducted to investigate other key metabolic enzymes involved in alternative carbon metabolism, notably those from gluconeogenesis and β-oxidation of fatty acids. Moreover, little is known about the transcriptional regulation and post-translation modification of these key metabolic enzymes in *C. glabrata*. Given the rapid emergence of invasive candidiasis caused by NCAC species, especially *C. glabrata*, it is envisaged that the knowledge generated from investigating this metabolic adaptation strategy will provide some basic insights into devising novel and innovative strategies for reducing the severity of invasive candidiasis caused by *C. glabrata* worldwide.

## Data Availability

Not applicable

## Abbreviations

BSIs: Bloodstream infections
CoA: Coenzyme A
CSRE: Carbon sources responsive element
DHAP: Dihydroxyacetone phosphate
GFP: Green fluorescent protein
ICU: Intensive care unit
NCAC: Non-*C. albicans Candida*
SCFAs: Short-chain fatty acids
SOT: Solid organ transplantation
TCA: Tricarboxylic acid cycle.
